# Feedback loops in intensive care unit prognostic models: an under-recognised threat to clinical validity

**DOI:** 10.1016/j.landig.2025.100880

**Published:** 2025-07-21

**Authors:** Daniel R Balcarcel, Sanjiv D Mehta, Celeste G Dixon, Charlotte Z Woods-Hill, Ewan C Goligher, Wouter A C van Amsterdam, Nadir Yehya

**Affiliations:** Division of Pediatric Critical Care Medicine, Department of Anesthesiology and Critical Care Medicine, Children’s Hospital of Philadelphia, Perelman School of Medicine, University of Pennsylvania, Philadelphia, PA, USA; Division of Pediatric Critical Care Medicine, Department of Anesthesiology and Critical Care Medicine, Children’s Hospital of Philadelphia, Perelman School of Medicine, University of Pennsylvania, Philadelphia, PA, USA; Division of Pediatric Critical Care Medicine, Department of Anesthesiology and Critical Care Medicine, Children’s Hospital of Philadelphia, Perelman School of Medicine, University of Pennsylvania, Philadelphia, PA, USA; Division of Pediatric Critical Care Medicine, Department of Anesthesiology and Critical Care Medicine, Children’s Hospital of Philadelphia, Perelman School of Medicine, University of Pennsylvania, Philadelphia, PA, USA; Interdepartmental Division of Critical Care Medicine, University of Toronto, Toronto, ON, Canada; Department of Data Science and Biostatistics, Julius Center for Health Sciences and Primary Care, University Medical Center Utrecht, Utrecht, Netherlands; Division of Pediatric Critical Care Medicine, Department of Anesthesiology and Critical Care Medicine, Children’s Hospital of Philadelphia, Perelman School of Medicine, University of Pennsylvania, Philadelphia, PA, USA

## Abstract

Prognostic models developed for use in the intensive care unit (ICU) can inform treatment decisions and improve patient care. However, despite extensive research, few models have contributed to improved patient-centred outcomes. A major limitation is that the influence of treatment interventions on patient outcomes during model development and validation is often overlooked. Upon implementation, prognostic models can affect clinical interventions, creating feedback loops that alter the relationship between predictors and observed patient outcomes. This alteration caused by model-mediated intervention is known as model drift. Positive feedback loops reinforce initial prognoses, leading to self-fulfilling prophecies, whereas negative feedback loops obscure the efficacy of successful interventions by rendering them as apparent model inaccuracies. To mitigate these issues, prognostic models for use in ICUs should account for treatment effects and the causal relationships among predictions, interventions, and outcomes. Thus, collaboration among data scientists, epidemiologists, clinical researchers, and implementation scientists is required to ensure that prognostic models enhance patient care without causing inadvertent harm.

## Introduction

Machine learning-based prognostic models have been developed and validated across a broad spectrum of tasks to support data-driven, patient-specific clinical interventions. These tasks include predicting future events, such as septic shock, acute kidney injury, acute respiratory distress syndrome, survival following extracorporeal membrane oxygenation (ECMO), and death.^1–5^ Many of these proposed models are highly accurate and expected to guide timely and patient-specific management decisions to improve overall outcomes. The intensive care unit (ICU), associated with high mortality and a vast amount of data availability, is an ideal setting for the application of prognostic models. However, despite their potential, the implementation of these models in health-care systems remains limited, and evidence of their contribution in improving outcomes is scarce.

The barriers to successful implementation are complex and multifactorial, and one causal factor might be that these models typically predict future physiological states, or their proxies, which can be altered by physician intervention during the interval between prediction and outcome. By influencing treatment decisions and thus modifying the actual outcome, prognostic models can have a causal effect on the system; this effect is challenging to incorporate into the model.^6,7^ These changes in decisions regarding the treatment regimen create feedback loops that fundamentally change the relationship between predictor variables (inputs) and observed patient outcomes, leading to misalignment with model predictions (outputs); this process, known as model drift, can compromise the performance of a model.

Harari, in the book *Sapiens: a brief history of humankind*, illustrates model drift by comparing two types of prediction systems.^8^ A level-one system is characterised by outcomes that are unaffected by predictions—an example being the weather; predicting rainfall does not influence the occurrence of actual precipitation. By contrast, predictions can affect the outcome in a level-two system. For example, a news report on a model forecasting an oil price increase of US$20 in the subsequent week could prompt immediate purchasing. This purchasing could drive the price beyond the initial forecast, making the actual future price unpredictable. Thus, in level-two systems, predictions themselves exert causal influence and alter the relationship between predictor variables and observed outcomes. Unless the model accounts for its own influence—an intrinsically complex task—the predictive power of the model remains low.^9–11^

Because prognostic models for use in the ICU are inherently designed to alter and improve patient outcomes, they operate predominantly as level-two systems and are inherently susceptible to model drift. For prediction models used in decision making, model drift can manifest as positive or negative feedback loops, as illustrated by the following examples.

## Positive feedback loops (self-fulfilling prophecies)

Positive feedback loops in prognostic models have been called self-fulfilling prophecies.^12,13^ A self-fulfilling prophecy occurs when the prediction of a model influences treatment decisions in a way that increases the likelihood of the predicted outcome over time, creating a self-amplifying source of bias. Such bias can occur when the model results are used to assign treatments on the basis of the risk of a predicted outcome.

**Figure 1:**
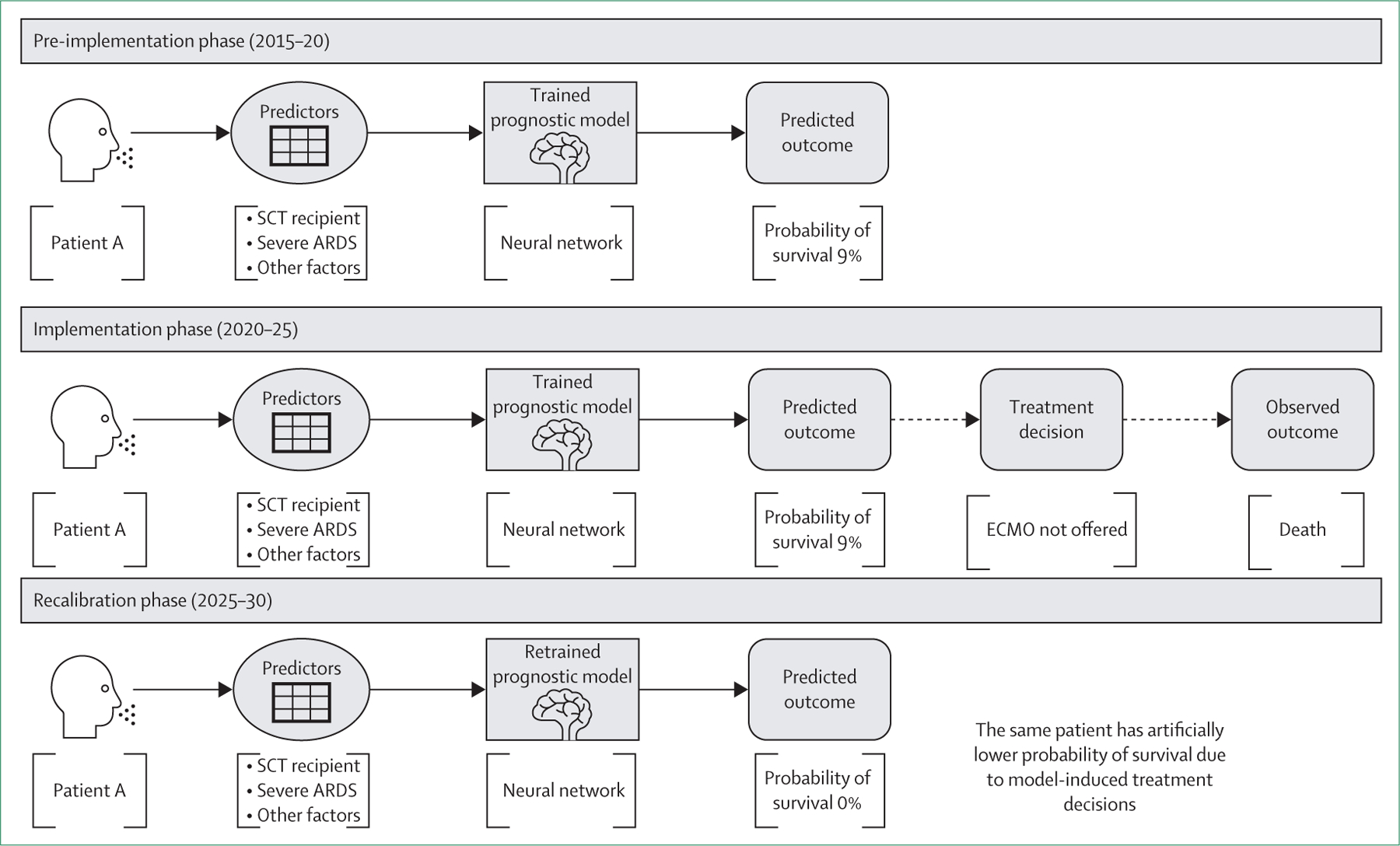
Positive feedback loop (self-fulfilling prophecy) Figure illustrates how prognostic models can create self-fulfilling prophecies by shaping treatment decisions in a way that reinforces their initial predictions. In the pre-implementation phase (2015–20), a model trained on historical data predicts a 9% survival probability for a patient with severe ARDS who has undergone HSCT. In the implementation phase (2020–25), this prediction guides treatment decisions, leading to the patient’s exclusion from ECMO owing to the predicted survival probability of less than 10%. Consequently, the patient does not receive ECMO and dies, validating the model’s prediction. When the model is retrained with updated data, it incorporates the biased outcomes it helped to create. Because patients who underwent HSCT were systematically denied ECMO, their observed survival rates decline further, resulting in a predicted survival probability of 0% in the recalibration phase (2025–30), perpetuating the cycle of exclusion and bias. ARDS=acute respiratory distress syndrome. ECMO=extracorporeal membrane oxygenation. HSCT=haematopoietic stem-cell transplantation. SCT=stem-cell transplantation.

Consider a model developed to assign venovenous ECMO candidacy on the basis of mortality risk (figure 1). The model was developed on the basis of the reasonable assumption that patients with a survival probability of less than 10% should not be offered venovenous ECMO, a resource-intensive therapy with a clinically significant complication rate. The model might predict that patients with severe acute respiratory distress syndrome and those who have undergone stem-cell transplantation have a survival probability of less than 10%. After implementation, the survival probability for patients who have undergone haematopoietic stem-cell transplantation (HSCT) might worsen owing to their systematic exclusion from venovenous ECMO, a potentially life-saving therapy. Although iterative improvements in ECMO technology could alter the risk profile for these patients over time, such changes would go unnoticed because of the rigid model implementation. Consequently, patients who could benefit from venovenous ECMO and technological advancements might face avoidable mortality.

In this example, a self-fulfilling prophecy arises because the prediction model simultaneously assigns treatments based on the risk of an outcome (survival) and biases the outcome towards its own prediction when clinicians act according to the model’s output. This clinical intervention leads to additional deaths among patients who have undergone HSCT. When models are updated (recalibrated) with new data, there is a risk of perpetuating the original assumption in a vicious self-amplifying cycle.

## Negative feedback loops (victim of its own success)

Feedback loops in outcome prediction models can also be negative. A negative feedback loop, often described as the model becoming a victim of its own success, occurs when an undesirable outcome is successfully prevented as clinicians intervene in response to a model prediction through targeted treatments.^10,14^

**Figure 2:**
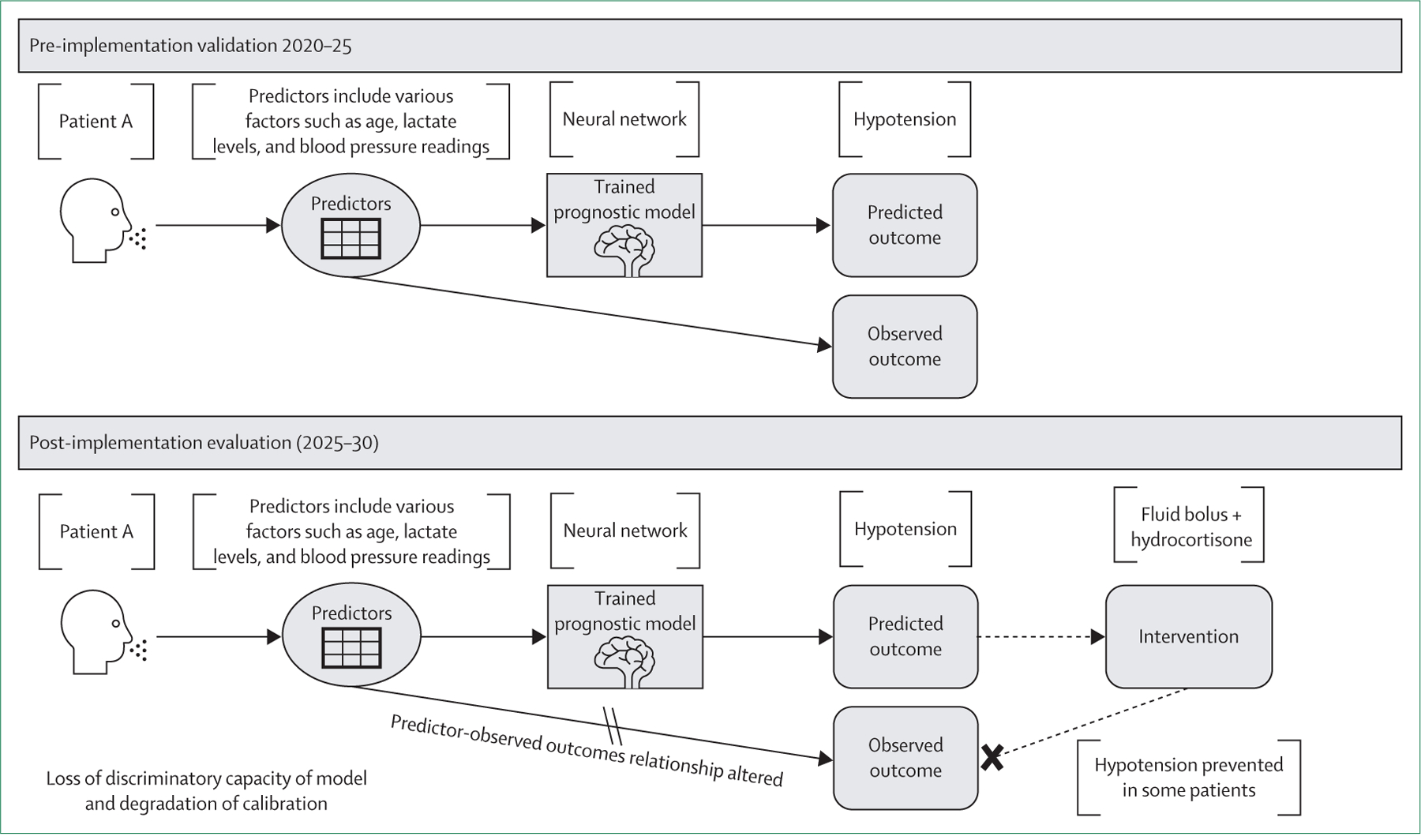
Negative feedback loop (victim of its own success) This figure illustrates how a prognostic model can become a victim of its own success. In the pre-implementation validation phase, the model is trained on historical data to predict the risk of hypotension in patients with sepsis within 6 h. It identifies patients at high risk with a high probability of hypotension and performs well. In the post-implementation evaluation phase, clinicians act on the model’s predictions by administering early interventions such as fluid boluses and hydrocortisone, successfully preventing hypotension. Therefore, the observed incidence of hypotension among patients at high risk declines. Although the model recommended preventive treatment, its predictions appear to overestimate risk, leading to a perceived decline in discrimination capacity and calibration.

Consider a septic shock prediction model that forecasts hypotension occurring within the next 6 h for patients with sepsis in the general ward (figure 2). After observing a prediction for a patient with a high probability of hypotension, clinicians might intervene by administering immediate fluid boluses and hydrocortisone. If these interventions are successful and hypotension is prevented, the predicted outcome (hypotension) might not occur. Consequently, when the model is evaluated after implementation, the observed incidence of hypotension among patients who were originally at high risk might be lower than expected—not necessarily because the model over-estimated the risk, but because the predictions prompted timely interventions that improved patient outcomes. This observation creates the illusion that the model over-estimates risk, making its performance appear worse than in pre-implementation (pre-intervention) validation studies. The issue arises because, during standard model evaluation, predicted probabilities are typically compared with observed outcomes without consistently accounting for the influence of clinical interventions. Updating and recalibrating the model becomes challenging without adjusting for treatment effects, because the relationships between predictors and observed outcomes have been fundamentally altered by model-induced clinician actions.

In negative feedback loops, models accompanied by the most successful interventions might paradoxically have the lowest accuracy after implementation.^14^ This process occurs because successful interventions reduce the occurrence of the predicted adverse event, resulting in an apparent overestimation of risk by the model. If standard model evaluation does not account for the effects of such interventions, observed outcomes after implementation will diverge from predicted probabilities, potentially undermining clinician trust in the model and discouraging its continued use—despite its actual benefit in preventing adverse outcomes. Moreover, as the model predictions become less aligned with observed outcomes because of the increased use of effective interventions, refining and recalibrating the model becomes increasingly challenging, potentially stalling further improvements. Addressing this issue requires model evaluation approaches that account for treatment effects.

## Addressing feedback loops and model drift

**Figure 3:**
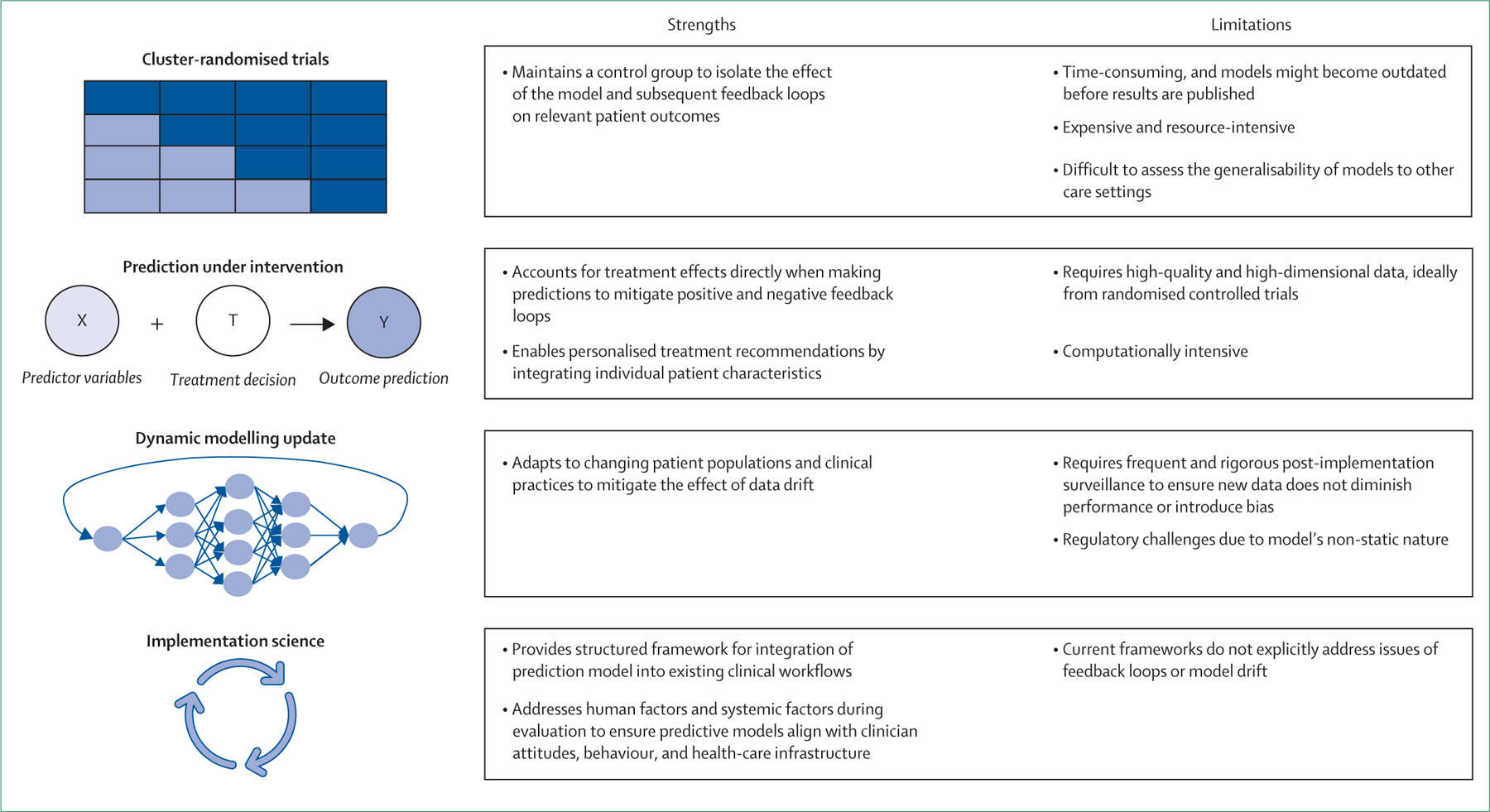
Tools for addressing feedback loops

Prognostic models can substantially improve the quality of care provided to patients. However, the current practice of evaluating prognostic models on the basis of observational data does not account for the causal effect exerted by these models after implementation, particularly when accompanied by treatment policies or decisions that are not included in the model. Addressing these challenges requires a multifaceted, interdisciplinary approach. Depending on the clinical context, various methodological approaches can be used to mitigate feedback loops and model drift, with some being more appropriate than others in any given scenario (figure 3). Because no single solution applies universally, researchers and clinicians should select and apply the tools that best address these challenges for each specific context.^15^

### Cluster-randomised trials

Prediction models might benefit from evaluation in a manner similar to traditional therapy evaluation, specifically through randomised controlled trials (RCTs).^6^ This evaluation could involve cluster-randomised trials, in which specific units, hospitals, or teams are randomly assigned to either implementation or non-implementation of the model to assess clinically significant outcomes across cohorts. This approach isolates the true influence of the model’s predictions on clinical decisions and patient outcomes, clarifying its benefits. Such an approach is in contrast with the objective of many stakeholders to rapidly implement these models and embed them into existing electronic health records, implicitly and presumptuously suggesting that no risks are associated with model implementation.

Although RCTs remain the gold standard for assessing interventions, their application for prognostic model evaluation presents several challenges. RCTs are often costly, logistically complex, or unethical.^6^ Moreover, the time required for planning, enrolment, data collection, analysis, and publication can span several years. During this period, shifts in patient demographics, clinical practice, and risk factors might render the model outdated or necessitate recalibration and retesting. Additionally, differences in patient populations and clinical practices across medical centres raise concerns about translatability of results, leading some researchers to question the broader generalisability of these models.^15–17^ These limitations render RCTs impractical for the continuous evolution of prognostic models, thus highlighting the need for alternative approaches to model development and evaluation.

### Prediction under intervention

Traditional prognostic models (ie, treatment-naive models) forecast patient trajectories without explicitly accounting for how their prediction influences treatment decisions. By contrast, the prediction under intervention framework takes a causally informed approach, estimating what a patient’s outcome would be under a specific treatment strategy when adjusting for individual characteristics.^6,18^ These models leverage causal inference techniques, such as G-computation, inverse probability weighting, and marginal structural models, to estimate counterfactual outcomes. Thereafter, these outcomes can be used to guide individualised treatment decisions.^19^ Although prediction under intervention models are ideally trained using RCT data, observational data can also be used once they are properly adjusted for confounders.^20^

These models have advantages as they account for treatment effects. First, they mitigate treatment-induced model drift, preventing feedback loops in which clinician responses to predictions inadvertently distort future outcomes. Second, they enable personalised treatment recommendations by integrating patient characteristics with intervention effects, thus optimising decision making at an individual level. Broadly, prediction under intervention models represent a fundamental shift towards causality-aware artificial intelligence, ensuring that prediction models do not simply forecast outcomes but actively influence better clinical decisions.

Despite these advantages, prediction under intervention models are not always feasible because they require high-quality, high-dimensional data, ideally from RCTs. When using observational data, these models require strong assumptions regarding unmeasured confounding, which might not always be valid or generalisable across different care settings and patient populations.^18^ Additionally, the complexity of causal inference techniques can escalate the computational requirements for model implementation, challenging implementation in real-time decision support systems. Further research is required to refine these approaches and assess their clinical influence at scale.

### Dynamic model updating

To maintain accuracy and utility in the rapidly changing health-care landscape, prognostic models should be subjected to continuous monitoring, validation, and improvement after implementation.^21^ Static models, even when proven to benefit patients, cannot consistently improve patient outcomes, as both patient populations and clinical practices evolve. The learning health system framework provides a structured approach to update a model by integrating local, real-world clinical data into iterative model refinement.^22^ In a learning health system framework, prediction models are not static but continually assessed and adjusted based on new evidence, ensuring that they remain aligned with best practices.

Unfortunately, evidence, guidance, and established frameworks for best practices for updating dynamic models are scarce.^21^ Although frequent updates might enhance predictive accuracy, they carry risks of introducing new biases or unintended consequences if not carefully evaluated. To mitigate such risks, collaboration between artificial intelligence researchers and causal inference experts is essential. Such partnerships can help to ensure that model updates enhance decision making and preserve reliability and fairness.

### Treatment-naive models

The final, least desirable, approach is to create treatment-naive prognostic models and hope that the issues of feedback loops are not substantial enough to negatively affect outcomes. Besides the disadvantage of introducing feedback loops, treatment-naive models assume a monotonous relationship between risk and benefit of treatment, which might not always be the case. In the ECMO example, a high risk of mortality might indeed imply futility of ECMO for some patients (eg, end-stage heart failure in a patient who is not a candidate for heart transplant), whereas for other patients, such as those who have undergone transplantation, ECMO might be beneficial, despite a high risk of death.

Prognostic models used solely for risk assessment or risk stratification should be distinguished from those that inform treatment decisions. If a model is used only to estimate the probability of an event or to identify risk factors that can be acted upon, causal inference might not be necessary. However, feedback loops can arise when a prognostic model directly or indirectly influences treatment decisions, especially in settings with a considerable time gap between prediction and outcome. In such cases, understanding the causal relationship between predicted risks and treatment effects becomes essential. Although treatment-naive models might be suitable, they are far from ideal and might not improve patient outcomes or could lead to unintended consequences that negatively affect patients.

## Implementation science: a missing partner

Another crucial but often overlooked aspect of predictive model development is the integration of implementation science. This field focuses on embedding evidence-based interventions into routine clinical practice to sustainably improve patient outcomes.^23^ Although considerable efforts have gone into model development and validation, less attention has been given to preparing health-care systems and clinicians for their real-world implementation.^24^ Simulation-based evaluation frameworks showed that a model’s clinical utility is determined not solely by its predictive accuracy but also by how well it aligns with provider workflows, intervention timing, and resource availability.^25^ This disconnect between the focus on predictive accuracy and practical, real-world factors persists despite growing recognition that the success of prognostic models hinges on more than simply achieving impressive accuracy metrics, such as the area under the receiver operating characteristic curve.^26^

A validated, effective model still requires appropriate implementation to improve outcomes. Anticipating and addressing barriers to model adoption early in the process ensures that predictive models are not only theoretically sound but also practically viable. Clinician adherence to model recommendations is just as essential as model performance in finding out its clinical utility. For instance, if a model predicts acute kidney injury and recommends interventions, measuring how consistently clinicians follow those recommendations can clarify why a model appears less accurate over time (negative feedback loop). In cases where adherence to model-suggested recommendations is high, perceived poor performance (victim of its own success) could signal success, warranting broader use rather than abandonment. Preparing health-care systems for implementation and continuous evaluation of model performance after integration of treatment decisions should be essential steps, requiring intentional and sustained collaboration with implementation scientists.

## The road ahead

Substantial efforts are under way to develop prognostic models; however, few account for model-induced treatment effects and subsequent risk of feedback loops. The success of these models will depend not only on their ability to incorporate treatment decisions but also on their capacity to dynamically adapt to clinical environments. However, this evolving nature also presents a major unaddressed regulatory challenge. Traditional regulatory frameworks for medical technology assume a fixed product, whereas prognostic models that incorporate adaptive learning are inherently designed to change over time. Regulators will need to not only evaluate a model’s initial validity and performance but also continually monitor its safety, fairness, and performance as it evolves with changing clinical ecosystems. Unlike traditional medical devices, these models continuously interact with and reshape clinical practice, making pre-implementation approval insufficient. Frequent and rigorous post-implementation surveillance is required.

Addressing this challenge will require regulatory innovation, which includes explicit guidelines. Current guidelines, such as TRIPOD+AI, do not mandate reporting of how model predictions and subsequent treatments might influence performance and utility after implementation.^27^ Future regulatory pathways and guidelines should incorporate real-time model auditing, periodic recalibration requirements, and post-deployment surveillance to mitigate risks associated with feedback loops and unintended consequences. These approaches are crucial in ensuring the safety and utility of adaptive models in evolving health-care settings.

## Conclusion

To enhance the clinical utility of prognostic models in health-care systems, the influence of treatment decisions informed by these models on both model output and patient outcomes should be considered. Adopting this perspective necessitates a paradigm shift in the development, validation, and implementation of prognostic models that includes explicit treatment policies and causal thinking. Ultimately, this will require collaboration from a broad range of experts, including data scientists, epidemiologists, clinical researchers, and implementation scientists.
